# Hepatitis B virus surface antigen seroconversion in HIV-infected individual after pegylated interferon-alpha treatment: a case report

**DOI:** 10.1186/1678-9199-19-31

**Published:** 2013-12-10

**Authors:** Adriane Maira Delicio, Paulo Afonso Martins Abati, Aline Gonzalez Vigani

**Affiliations:** 1 Campinas Reference Center for Sexually Transmitted Diseases/AIDS, Campinas, São Paulo State, Brazil; 2 Hepatitis Virus Study Group, University of Campinas (UNICAMP), Campinas, São Paulo State, Brazil

**Keywords:** HIV, Hepatitis B, HBsAg, Peginterferon

## Abstract

Hepatitis B virus (HBV) infects from 6 to 14% of HIV-infected individuals. Concurrent HIV/HBV infection occurs due to the overlapping routes of transmission, particularly sexual and parenteral. HIV-infected patients that have acute hepatitis B have six times greater risk of developing chronic hepatitis B, with higher viral replication, rapid progression to end-stage liver disease and shorter survival. The coinfection is also associated with poor response to hepatitis B treatment with interferon-alpha and increased liver toxicity to the antiretroviral therapy. Herein, we describe the case of a 35-year-old man who engages in sex with men and presented with newly diagnosed HIV-1, serological markers for acute hepatitis B and progression to chronic hepatitis B infection (HBsAg+ > 6 months, high alanine aminotransferase levels and moderate hepatitis as indicated by liver biopsy). Lacking indication of antiretroviral treatment (CD4 768 cells/mm^3^), he was treated with pegylated-interferon alpha2b (1.5 mg/kg/week) by subcutaneous injection for 48 weeks. Twelve weeks after treatment, the patient presented HBeAg seroconversion to anti-HBe. At the end of 48 weeks, he presented HBsAg seroconversion to anti-HBs. One year after treatment, the patient maintained sustained virological response (undetectable HBV-DNA). The initiation of antiretroviral therapy with nucleosides and nucleotides is recommended earlier for coinfected individuals. However, this report emphasizes that pegylated interferon remains an important therapeutic strategy to be considered for selected patients, in whom the initiation of HAART may be delayed.

## Background

Worldwide, an estimated two billion people have been infected with the hepatitis B virus (HBV), more than 350 million have chronic liver infection and 34 million live with the human immunodeficiency virus (HIV) infection [1,2]. The prevalence of hepatitis B in HIV-infected individuals ranges from 6 to 14%, with higher rates in regions where HBV is endemic, such as Africa and Asia [1,3]. Concurrent infection of HIV with HBV occurs because of the overlapping routes of transmission, particularly sexual and parenteral (injecting drug use and blood transfusion).

In Brazil, the prevalence of coinfection ranges from 5.3 to 24.3% with significant regional differences [4]. The western Amazon is considered highly endemic for hepatitis B, in particular because of the coinfection with hepatitis Delta virus (HDV) [1]. In addition, a recent population-based prevalence study in Brazilian state capitals showed that all geographical regions were considered of low endemicity [5]. Acute hepatitis B patients with HIV infection have six times greater risk of developing chronic hepatitis B, with higher viral replication, rapid progression to end-stage liver disease and shorter survival [6]. The coinfection is also associated with poor response to hepatitis B treatment with interferon-alpha, lower rates of HBeAg seroconversion to anti-HBe and HBsAg to anti-HBs, and greater liver toxicity using the antiretroviral therapy [7-10]. Since the introduction of highly active antiretroviral therapy (HAART), liver disease has emerged as an important cause of morbidity and mortality in HIV-infected individuals. Treatment of chronic hepatitis B is based on the use of pegylated interferon or nucleosides and nucleotides polymerase inhibitors. The international guidelines recommend the use of pegylated interferon for the treatment of HBeAg positive coinfected patients that are non-cirrhotic and with no indication for antiretroviral therapy, with limited treatment duration and probably greater chance of viral suppression after discontinuation [11,12].

## Case presentation

We report the case of a 35-year-old male patient who engages in sexual activity with men, followed in the Campinas Reference Center for Sexually Transmitted Diseases/AIDS since January 2009, with newly diagnosed HIV-1 and the following serological markers for hepatitis B: surface antigen positive (HBsAg+), hepatitis B *e* antigen positive (HBeAg+), antibodies to hepatitis B core antigen positive (anti-HBc IgM+, anti-HBc IgG+), anti-hepatitis B *e* antibody negative (anti-HBe-), and anti-hepatitis B surface antigen antibody negative (anti-HBs-). He had a steady partner who presented a diagnosis of hepatitis B with seroconversion to anti-HBs + about four months before the start of the monitoring service and HIV-1 negative at diagnosis of the patient, with seroconversion to HIV-1 positive in 2010.

On physical examination, the patient was in good general condition and his body mass index was 24 kg/m^2^. His liver was palpable at 2 cm from the right costal margin and his spleen was palpable at 2 cm from the left costal margin. He had no stigmata of chronic liver disease.

In February 2009, CD4+ lymphocyte (CD4) count was 718 cells/mm^3^, HIV-1 viral load (VL) was 9399 copies/mL, aspartate aminotransferase (AST) was 347 IU/mL, alanine aminotransferase (ALT) 604 IU/mL, HBsAg+, HBeAg+, anti-HBc IgG+, anti-HBc IgM+, anti-HBe-, anti-HBs-, antibody for hepatitis C virus (HCV) negative, and antibody for hepatitis A (HAV) IgG+, IgM- (Table 1). The biochemical liver function test values were normal. Upper endoscopy showed gastroduodenitis with *H. pylori* positive and total abdominal ultrasound showed no abnormality.

**Table 1:** Laboratory tests performed during follow-up

**Laboratory tests**	**Date**						
	**Feb. 16, 2009**	**Oct. 13, 2009**	**May 13, 2010**	**Jul. 22, 2010**	**Feb. 21, 2011**	**Jun. 9, 2011**	**May 5, 2012**
CD4 (cells/mm^3^)	718	768	678	520	635	597	466
HIV viral load (copies/mL)	9399	3746	2553	79	448	2536	–
Hemoglobin (g/dL)	16.7	15.2	15.4	14.6	14.0	15.5	–
Neutrophils/mL	2236	1867	714	2436	3129	2217	–
Platelets 10^3^/mL	192	187	124	146	173	186	–
AST/ALT (IU/mL)	347/604	101/136	217/223	31/31	24/14	26/16	26/14
HBsAg	Positive	Positive	–	Positive	Negative	Negative	Negative
HBeAg	Positive	Positive	–	Negative	–	–	–
Anti-HBc IgG	Positive	Positive	–	Positive	Positive	Positive	Positive
Anti-HBc IgM	Positive	Negative	–	Negative	–	–	–
Anti-HBe	Negative	Negative	–	Positive	–	–	–
Anti-HBs	Negative	Negative	–	Negative	> 1000	716	148

The patient was classified into the first stage of HIV infection (A1), according to the Centers for Disease Control and Prevention (CDC) criteria and acute HBV infection. Then, it was decided to monitor the hepatitis B progress and not initiate antiretroviral therapy.

After eight months of follow-up, the patient had the following laboratory tests results: CD4 768 cells/mm^3^, VL 3746 copies/mL, AST 101 IU/mL, ALT 136 IU/mL, HBsAg+, HBeAg+, anti-HBc IgG+, anti-HBc IgM-, anti-HBe-, and anti-HBs- (Table 1).

When chronic hepatitis B (HBsAg+ > 6 months) was confirmed, a liver biopsy was performed resulting in METAVIR score F2A2. Because of the persistence of HBsAg, increased ALT levels and no indication of antiretroviral therapy, treatment was initiated for hepatitis B with pegylated-interferon alpha2b (80 mcg), 1.5 mg/kg once a week, by subcutaneous injection for 48 weeks.

**Figure 1:**
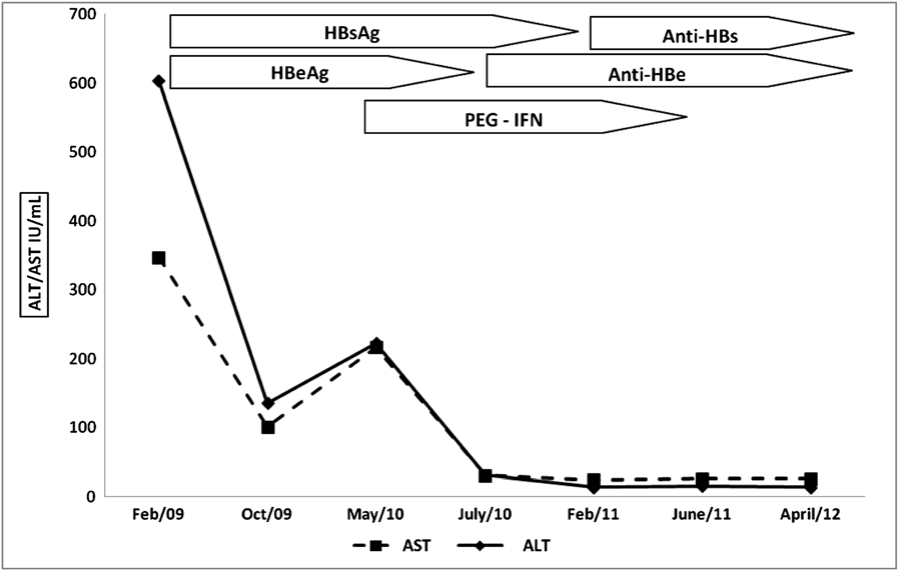
Evolution of biochemical and serological tests before and after treatment.

During treatment, the patient developed moderate neutropenia (minimum of 714 neutrophils/mL) controlled by use of filgastrima, thrombocytopenia (minimum of 124,000 platelets/mL), nadir CD4 520 cells/mm^3^ and bipolar affective disorder, stabilized on lithium carbonate (Table 1). At 12 weeks of treatment, the patient presented HBeAg seroconversion to antiHBe and normalization of liver enzymes. At the end of 48 weeks of treatment, he presented HBsAg-, anti-HBc IgG+, anti-HBs > 1000 mIU/mL (Figure 1). In May 2012, a year after the end of treatment, the patient showed AST 26 IU/mL, ALT 14 IU/mL, anti-HBc IgG+, anti-HBs 148, CD4 466 cells/mm^3^ and HBV-DNA level < 15 IU/mL (reference values 15 – 500.000.000 IU/mL by real time PCR).

## Discussion

This report describes the case of a HIV/HBV-coinfected patient, without indication of antiretroviral therapy, who had optimal response to treatment of chronic hepatitis B with pegylated interferon. The patient presented rapid HBeAg seroconversion to anti-HBe and HBsAg to anti-HBs and sustained virological response one year after the end of therapy.

According to the literature, HBeAg seroconversion to anti-HBe occurs in up to 30% of monoinfected individuals in use of pegylated interferon and in up to 28% of coinfected individuals in use of conventional interferon-alpha [8,11,13,14]. Furthermore, HBsAg seroconversion to anti-HBs occurs in less than 10% of the monoinfected and probably in a much smaller proportion of coinfected individuals [15]. However, there are no published studies that evaluate the therapeutic response to pegylated interferon in a representative group of coinfected individuals.

There are few studies showing HBeAg and/or HBsAg seroconversion in coinfected individuals, whether on HAART (nucleoside and nucleotide polymerase inhibitors) or with use of interferon-alpha [16]. In the early 1990s, the first case reporting HBsAg seroconversion in HIV/HBV-coinfected patients treated with conventional interferon-alpha was published [17]. Later, it was reported a case of HBsAg seroconversion in a HIV/HBV/HCV/HDV-coinfected patient after treatment with pegylated interferon and ribavirin [18]. The most recent report of hepatitis B surface antigen seroconversion in a HIV/HBV-coinfected patient treated with pegylated interferon was described by Rossetti *et al*. [19] in 2012, with some similarities to our patient.

The lower probability of spontaneous loss of HBeAg and HBsAg in HIV/HVB-coinfected individuals, as well as lower seroconversion after treatment, may be explained by the impairment of innate and adaptive immunity, with a weak cellular immune response to HBV, typical of HIV-infected patients [20,21]. As a cytokine, interferon-alpha acts inhibiting HBV replication through interaction with viral proteins and stimulation of host cellular immunity. Moreover, it is supposed that the best therapeutic response in HIV/HBV-coinfected patients – avoiding antiretroviral therapy and in non-cirrhotic individuals – may be obtained in cases of HBeAg positive, with higher CD4 counts, lower HBV-DNA levels, higher ALT levels, early stages of liver fibrosis and genotype A [20-24]. Although some authors suggest the importance of quantitative measurement of HBsAg as predictor of virological response and HBsAg seroconversion in monoinfected individuals, it has not been proven yet in HIV/HBV-coinfected individuals [25,26]. It is known that HIV infects hepatocytes already infected with HBV and leads to increased intracellular levels of HBsAg, which could contribute to accelerated liver disease in these individuals. HBsAg levels could not be quantified in this study [27].

## Conclusion

Currently, the initiation of antiretroviral therapy with nucleosides and nucleotides is recommended earlier for coinfected individuals, even in patients with higher CD4 counts compared to HIV-monoinfected patients [11,12,28,29]. However, the pegylated interferon remains an important therapeutic strategy to be considered for selected patients, in whom the initiation of HAART may be delayed, aiming at a more effective and lasting response to treatment, with lower risk of progression to cirrhosis and hepatocellular carcinoma, as the example of the patient described in the present study [30].

## Consent

Written informed consent was obtained from the patient for publication of this case report.

## Abbreviations

ALT: Alanine aminotransferase; Anti-HBc: Antibodies to hepatitis B core antigen; Anti-HBe: Anti-hepatitis B *e* antibody; Anti-HBs: Anti-hepatitis B surface antigen antibody; AST: Aspartate aminotransferase; CDC: Centers for disease control and prevention; CD4: CD4+ lymphocyte; DNA: Deoxy ribonucleic acid; HAART: Highly active antiretroviral therapy; HAV: Hepatitis A virus; HBV: Hepatitis B virus; HbsAg: Hepatitis B surface antigen; HBeAg: Hepatitis B *e* antigen; HCV: Hepatitis C virus; HDV: Hepatitis Delta virus; HIV: Human immunodeficiency virus; VL: Viral load; PCR: Polymerase chain reaction.

## Competing interests

The authors declare that they have no competing interests.

## Authors’ contributions

AMD was the responsible doctor for the patient’s treatment and conceived the study. AMD and PAMA wrote the literature review and organized the manuscript. AGV reviewed the article. All authors read and approved the final text.
